# A Melanoma Molecular Disease Model

**DOI:** 10.1371/journal.pone.0018257

**Published:** 2011-03-30

**Authors:** Smruti J. Vidwans, Keith T. Flaherty, David E. Fisher, Jay M. Tenenbaum, Michael D. Travers, Jeff Shrager

**Affiliations:** 1 CollabRx Inc., Palo Alto, California, United States of America; 2 Massachusetts General Hospital Cancer Center, Boston, Massachusetts, United States of America; 3 Department of Dermatology, Cutaneous Biology Research Center and Melanoma Program, Massachusetts General Hospital, Boston, Massachusetts, United States of America; 4 Symbolic Systems Program (Consulting), Stanford University, Stanford, California, United States of America; King Abdullah University of Science and Technology, Saudi Arabia

## Abstract

While advanced melanoma remains one of the most challenging cancers, recent developments in our understanding of the molecular drivers of this disease have uncovered exciting opportunities to guide personalized therapeutic decisions. Genetic analyses of melanoma have uncovered several key molecular pathways that are involved in disease onset and progression, as well as prognosis. These advances now make it possible to create a “Molecular Disease Model” (MDM) for melanoma that classifies individual tumors into molecular subtypes (in contrast to traditional histological subtypes), with proposed treatment guidelines for each subtype including specific assays, drugs, and clinical trials. This paper describes such a Melanoma Molecular Disease Model reflecting the latest scientific, clinical, and technological advances.

## Introduction

Melanoma is the most aggressive form of skin cancer and its incidence is on the rise worldwide [1]. While early stages of melanoma can be successfully treated by surgical excision, advanced stages are uniquely refractory to current therapies. However, we now recognize that melanomas are far more variable at a molecular level than they appear under the microscope. Therefore, rather than treating melanoma as a single disease, it makes sense to stratify tumors into molecular subtypes and treat each with the most appropriate therapies. This approach is supported by the dramatic success of PLX4032 for melanoma tumors possessing the BRAF V600E mutation [2], and Imatinib for those possessing C-KIT mutations [3]–[5].

With hundreds of molecular diagnostics and targeted therapies in development, the time is ripe to develop a formal process for classifying melanoma into molecular subtypes, and for developing proposed treatment guidelines for each subtype, including specific assays, drugs, and clinical trials. This process produces a formal 'Molecular Disease Model' (MDM) that can be used by clinicians to guide treatment decisions, and refined by researchers based on clinical outcomes and laboratory findings.

**Figure 1:**
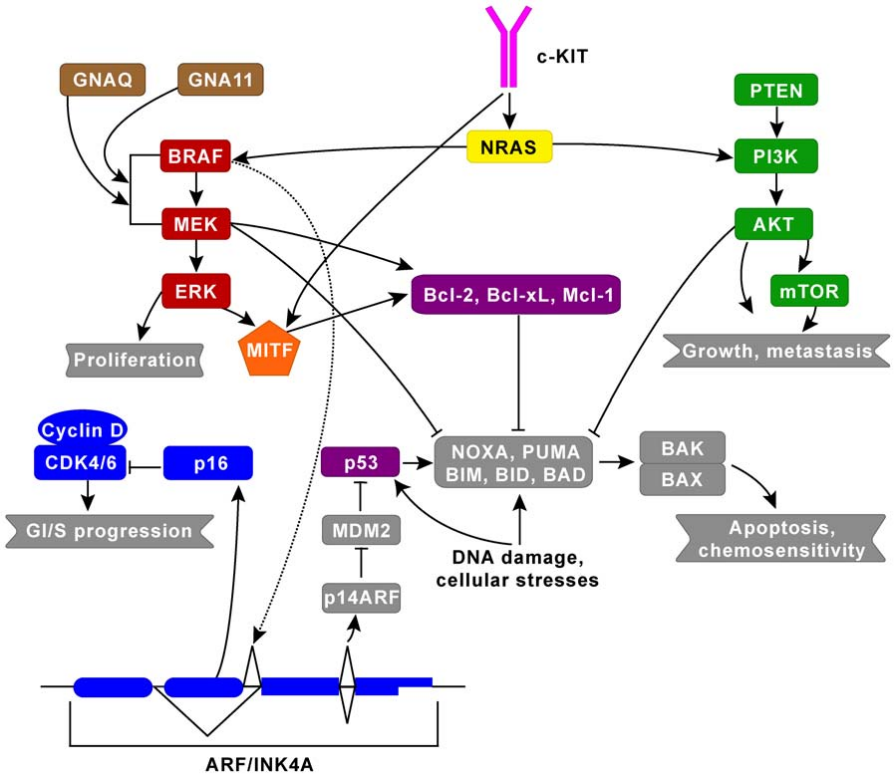
The two major signaling pathways implicated in melanoma are the MAPK pathway (red) and the AKT/PI3K (green) pathway which regulate cell growth, proliferation and cell death. There is a lot of cross-talk between these pathways and their downstream effectors, which we have classified into 8 pathways for simplicity to account for differences in treatment modalities (e.g. signaling through NRAS could affect both MAPK and AKT/PI3K pathways). The additional 6 pathways are: c-KIT (pink), CDK (blue), GNAQ/GNA11 (brown), MITF (orange), NRAS (yellow), and P53/BCL (purple). The complex relationship among BRAF, ARF/INK4A (via dashed line), p16, and p14ARF connotes an alternative splicing relationship.

This paper outlines such a Molecular Disease Model for melanoma. The model consists of a set of actionable molecular subtypes and proposed practice guidelines for treating each subtype: which therapies (approved or experimental) should be considered and which are contraindicated (see Tables 1 and 2). A molecular subtype of melanoma is loosely defined as those tumors containing the same set of molecular (primarily genetic) defect(s) and their associated pathways (see Figure 1). A subtype is deemed actionable if there is both a CLIA-approved assay to determine whether a given tumor fits that classification, and at least one FDA-approved or experimental targeted therapy with potential efficacy for that subtype. An example would be melanoma tumors containing a BRAF V600E mutation for which commercial assays and targeted agents are currently available. The latest version of the Melanoma Molecular Disease Model can be found online here: http://mmdm.cancercommons.org/smw/index.php/A_Melanoma_Molecular_Disease_Model.

**Table 1:** Principal melanoma molecular subtypes.

Detailed subtypes	Pathway(s)	Key gene /biomarker(s)	Diagnostic technologies	Potentially relevant therapeutics
1.1	MAPK	BRAF	Targeted sequencing	BRAF inhibitors, MEK inhibitors, Hsp90 inhibitors
1.2		BRAF/PTEN	Targeted sequencing & IHC	(BRAF inhibitors) AND (PI3K inhibitors, AKT inhibitors or mTOR inhibitors)
1.3		BRAF/AKT	Targeted sequencing & copy number	(BRAF inhibitors) AND (AKT inhibitors or mTOR inhibitors)
1.4		BRAF/CDK4	Targeted sequencing & copy number/CGH	BRAF inhibitorsAND CDK inhibitors
2.1	c-KIT	c-KIT	Targeted sequencing	Gleevec & other c-KIT inhibitors
3.1	GNAQ GNA11	GNAQ	Targeted sequencing	MEK inhibitors
3.2		GNA11	Targeted sequencing	MEK inhibitors
4.1	NRAS	NRAS	Targeted sequencing	MAPK & PI3K pathway inhibitors; Farnesyl transferase inhibitors
5.1	MITF	MITF	Copy number	HDAC inhibitors

**Table 2:** Secondary melanoma molecular subtypes.

Detailed subtypes	Pathway(s)	Key gene /biomarker(s)	Diagnostic technologies	Potentially relevant therapeutics
6.1	AKT/PI3K	PTEN	IHC	PI3K inhibitors, AKT inhibitors or mTOR inhibitors
6.2		AKT	Copy number	AKT inhibitors or mTOR inhibitors
6.3		PI3K	IHC	PI3K inhibitors, AKT inhibitors or mTOR inhibitors
7.1	CDK	ARF/INK4A	Targeted sequencing / CGH	CDK inhibitors
7.2		CDK4	Copy number / CGH	CDK inhibitors
7.3		CCND1 / Cyclin D1	Copy number / CGH	CDK inhibitors
8.1	P53 / BCL	Bcl-2	IHC	TBD
8.2		P53	Targeted sequencing	TBD

The online version contains additional in-depth information about relevant genes, genetic tests, pathways, drugs, targets, and clinical trials, all hyperlinked and organized in a Wikipedia-like format. Given the evolving state of knowledge, we anticipate that this baseline model will need to be revised with new clinical and scientific findings. Existing types are likely to be split into new subtypes corresponding to responders and non-responders, and new types are likely to be added to accommodate previously unseen tumor types. Over time, this model will be defined with greater and greater specificity and linked to increasingly efficacious therapies.

## Methods

The initial subtypes and associated practice guidelines defined here were identified by consensus of a panel of recognized melanoma experts, and supported by detailed analysis of the peer-reviewed scientific literature. Subtypes are defined based on the status of key melanoma genes/pathways and their combinations. Each subtype is defined by one key oncogene/tumor suppressor (such as BRAF for subtypes 1.1 to 1.4, and c-KIT for subtype 2.1), either by itself or in combination with others that play a supportive role (such as PTEN, AKT and CDK4 in the case of subtypes 1.2, 1.3 and 1.4).

## Results

Tables 1 and 2 summarize the types of melanoma, roughly in order of importance of the associated oncogene/tumor suppressor, prevalence and potential for therapeutic intervention. We believe that the oncogenes that define the subtypes in Table 1 are capable of serving as the dominant oncogene and putative point of intervention for therapy, whereas the oncogenes and tumor suppressor genes that define subtypes in Table 2 play a supportive role and typically co-exist with the mutations outlined in Table 1.

Table S1 provides the drugs, their manufacturer and their putative targets for each subtype, as well as the relevant clinical trials and their status. It is our hope that the present model serves to focus translational research on issues that may directly impact patient care, and that the resulting activity will lead to updates in the model. For example, melanomas that fit into one of these subtypes, but which do not respond as predicted, may necessitate splitting of that subtype in a future revision of the model.

### Subtype 1

Subtype 1 harbors aberrations in the MAPK (Mitogen-activated protein kinase) pathway, either by itself or in combination with others such as the AKT/PI3K and CDK pathways. The MAPK pathway is a phosphorylation-driven signal transduction cascade that couples intracellular responses to the binding of growth factors to cell surface receptors. This pathway regulates several processes including cell proliferation and differentiation, and is often dysregulated in a variety of cancers. The classical MAPK pathway consists of RAS, RAF, MEK and ERK, where RAS triggers the formation of a RAF/MEK/ERK kinase complex which then drives transcription of key regulators through protein phosphorylation. Each of these components is encoded by several genes that play subtly distinct roles in signal transduction. For example, the RAF kinase family consists of three members: ARAF, BRAF and CRAF each of which can activate MEK/ERK signaling [6]. Molecular tests associated with subtypes 1.1–1.4 include: BRAF targeted sequencing for the presence of V600E mutation, Immuno-Histo-Chemical (IHC) tests for reduced PTEN protein levels, tests examining increased copy number of AKT, and IHC indicating increased CCND1/Cyclin D protein levels.

### Subtype 1.1 overview

Subtype 1.1 is characterized by a mutation in the BRAF gene. BRAF encodes a serine/threonine-protein kinase and is the most commonly mutated gene in melanoma (observed to be mutated in 40–70% of melanoma) [7]. While >60 mutations have been mapped in BRAF, a valine to glutamic acid change at codon 600 (V600E) occurs in >90% of cases. This mutation leads to constitutive activation of BRAF by bypassing the need for activation by NRAS and ATP. In addition, this mutant protein is >10-fold more active than wildtype BRAF. Taken together, these data indicate the importance of BRAF as a therapeutic target in melanoma. In some melanomas, BRAF mutations occur along with other mutations in genes such as PTEN and CDK4. These double mutant combinations are described below. However, since melanomas are not routinely screened for these genes, some melanomas that belong to these subtypes may be mis-classified as 1.1.

### Potential therapeutic approach for subtype 1.1

There are several potential targets for therapeutic intervention in this pathway including BRAF, MEK, ERK and Hsp90. Drugs targeting BRAF, MEK, and Hsp90 (but not ERK) are in development, and clinical trials are ongoing to evaluate their efficacy in melanoma.

### BRAF inhibitors

The BRAF inhibitor, Sorafenib/Nexavar, was the first RAF kinase inhibitor to be tested in humans. It is a broad RAF kinase inhibitor that competes with ATP for binding to RAF. Sorafenib suppresses BRAF as well as CRAF with similar efficiency by stabilizing the inactive conformations, though it is less efficacious on the BRAF V600E form than on wildtype. Sorafenib failed to demonstrate efficacy against melanoma by itself but may be somewhat effective with chemotherapy, albeit independently of BRAF status.

Several second generation inhibitors with greater specificity than Sorafenib are in development and one of these, PLX4032, appears very promising. This drug is highly specific for the V600E version of BRAF. Results of a Phase I study announced in 2009 at ASCO's annual conference indicated a response in almost 80% of participants and tumor shrinkage was observed in nearly all patients [8]. Further testing is ongoing in patients with the BRAF V600E mutation.

Another exciting inhibitor of BRAF V600E is GSK2118436 which is a highly potent and selective ATP competitive BRAF inhibitor with >100-fold selectivity for mutant (mut) BRAF over wild type (wt) in cell lines. In a Phase I/II, clinical activity with minimal toxicity was observed at multiple dose levels in mutant BRAF tumors [9]. Arguably the most exciting characteristic of this drug is its potential to control brain metastases in melanoma patients, which are notoriously resistant to drug therapy. Of 10 trial participants with previously untreated brain metastases, all experienced control of melanoma brain metastases, and 9 of the 10 patients had reductions in the overall size of their brain metastases [10].

Other selective BRAF inhibitors in clinical testing include RAF265 (an inhibitor of ARAF, CRAF and mutant & wildtype BRAF) and XL281 (an inhibitor of CRAF and both wildtype and V600E BRAF). Results of a Phase I study of XL281 presented at ASCO's annual conference in 2009 showed clinical benefit in 43% of enrollees [11], however, some systemic toxicity was noticed that could hamper its treatment potential. RAF265 is currently being evaluated in the Phase I setting for melanoma.

While there is great hope that these drugs will successfully halt progress in patients with BRAF mutant melanomas, emerging data suggests that they might be counterproductive for patients with wildtype BRAF. Treatment of BRAF-wildtype cells with these inhibitors appears to induce the MAPK pathway through several mechanisms [12]–[14]. These results suggest that PLX4032 and others might be indicated only for patients whose tumors harbor activating mutations in BRAF.

### MEK inhibitors

The MEK inhibitor, AZD6244/ARRY-142886, is an ATP non-competitive, allosteric inhibitor of MEK1/MEK2. In a Phase II trial in melanoma, AZD6244 did not appear superior as compared to temozolomide. However, this trial was not restricted to patients with BRAF mutations [15]. Current studies in progress are selecting for patients based on their BRAF status. Another MEK1/2 inhibitor, GSK1120212, is in Phase II for BRAF-mutant melanoma as well as for melanomas with GNAQ and GNA11 mutations. Several others are also in Phases I and II for advanced malignancies and tumors.

### Hsp90 inhibitors

Hsp90 is a molecular chaperone that drives folding and stabilization of various cellular proteins, including BRAF. Hsp90 has preferential activity towards activated BRAF as compared to normal BRAF [16]. In addition, pharmacological inhibition of Hsp90 function results in degradation of activated BRAF, inhibition of MAPK activity and cell proliferation, and induction of apoptosis [17]. Several Hsp90 inhibitors are currently undergoing clinical investigation for melanoma.

### Subtypes 1.2 & 1.3 overview

These subtypes are characterized by abnormalities in both the MAPK and AKT/PI3K pathways. While current treatments and drug development programs are mostly centered on individual mutations and pathways, there is increasing evidence suggesting that melanoma requires multiple pathways for development and metastasis. The most widespread collaboration appears to be between the AKT/PI3K and MAPK pathways, specifically: 1. BRAF mutations are often accompanied by loss of PTEN or activation of AKT [18]–[19], 2. PTEN silencing is required for malignant transformation of BRAF-mutant melanoctyes in a mouse model [20], and 3. pharmacological inhibition of both, but not individual, pathways is highly effective in suppressing melanoma disease in pre-clinical models [21]–[25].

### Potential therapeutic approach for subtypes 1.2 & 1.3

There are several potential targets for therapeutic intervention for both pathways (i.e., BRAF and MEK for the MAPK pathway, and PI3K, AKT, and mTOR for the AKT/PI3K pathway). The overall approach for this subtype is combination therapy using inhibitors of each pathway. Several studies have been initiated or are in planning for dual inhibition of these pathways (see below). Specifically, NCT00349206 (M.D. Anderson Cancer Center) is testing Sorafenib plus Temsirolimus, and NCT01021748 (Merck & Astrazeneca) is testing MK2206 plus AZD6244.

### Subtype 1.4 overview

Subtype 1.4 is associated with aberrations in both the MAPK and CDK pathways, specifically activation of BRAF and over-expression of CCND1/Cyclin D. The CDK pathway has been suggested to contribute to metastasis of melanoma with BRAF mutations. Curtin and colleagues showed that primary melanomas arising from chronically sun-damages skin and mucosal sites, the latter of which typically do not harbor BRAF and NRAS mutations, have increased CCND1 copy number [26]. Unlike primary melanomas, >15% of metastatic melanoma samples with BRAF mutations exhibit amplification of CCND1. These melanomas are resistant to BRAF inhibitors highlighting the need for combination therapy [27].

### Potential therapeutic approach for subtype 1.4

The approach to treatment of subtype 1.4 is a combination of CDK and BRAF inhibitors. Several ongoing studies are investigating the impact of these individual classes of drugs on advanced melanoma, but none currently focus on both pathways concurrently. Given the role of CCND1 amplification in resistance to BRAF inhibitors, combination trials are likely on the horizon. In the meantime, a potential therapeutic approach is sequential administration of BRAF and CDK inhibitors, or perhaps even treatment with chemotherapeutic agents to potentially inhibit CDK/cell cycle pathways.

### Subtype 2

This subtype is characterized by mutations in the c-KIT pathway/complex, a receptor tyrosine kinase (RTK) that regulates intracellular processes such as cell growth, division, and migration in response to Stem Cell Factor (SCF) activity. c-KIT signal transduction appears to be at least partly mediated through activation of the MAPK, the AKT/PI3K, and/or the MITF pathways [28]–[29]. Activating c-KIT mutations have been implicated in a variety of cancers [30] starting with GIST (Gastrointestinal stromal tumors) and CML (Chronic Myelogenous Leukemia). Because of this there are both approved drugs and drugs in clinical development that target c-KIT including Imatinib, Sunitib, Nilotinib, and Dasatanib. Mutations in c-KIT exon 11 (L576P) and exon 13 can be detected by targeted sequencing.

### Subtype 2.1 overview

Currently 2.1 is the only subtype in this category and is characterized by genetic aberrations in c-KIT, typically including mutations and/or copy number increases [31]. In 2006, Bastian and colleagues conducted a systematic study evaluating the frequency of c-KIT aberrations in melanoma finding mutations and/or copy number increases in 39% of mucosal, 36% of acral, and 28% of melanomas on chronically sun-damaged skin, but not in melanomas on skin without chronic sun damage [32]. This finding is consistent with those of several additional studies that have also investigated the frequency of c-KIT aberrations [33]. However most of these studies have been conducted on small patient samples and larger numbers, as well as consecutive patient-studies, will be needed to determine more accurate incidences of c-KIT in various types of melanoma. c-KIT aberrations do not seem to typically overlap with mutations such as B-RAF and NRAS even though these are amongst the most common mutations in melanoma [32].

### Potential therapeutic approach for subtype 2.1

The general treatment strategy for subtype 2.1 is c-KIT inhibitors, several of which are either approved or in development (see below). In the early 2000 s, three Phase II clinical trials failed to show significant responsiveness of metastatic melanoma to Gleevec treatment, however patients in these trials were not selected on the basis of their c-KIT status. The only responder in this trial had very high KIT protein expression, supporting the hypothesis that c-KIT aberrant melanomas are responsive to c-KIT inhibitors such as Gleevec.

Additional support has come from individual case studies:

- A patient with anal mucosal melanoma and metastases to lymph nodes harbored an amplified KIT K642E mutation. Complete resolution of subcutaneous melanoma and nodules was achieved after a dose-escalation Gleevec regimen [34].
- A patient with KIT V560D mutant anal melanoma with isolated lung metastases had a complete response to a combination of Nexavar and Temozolomide [35].
- A patient with with a KIT PYDHKWE duplication rectal melanoma demonstrated a significant clinical response after 4 weeks of Gleevec treatment [36].
- A patient with a KIT L576P vaginal mucosal melanoma and extensive metastases to lymph nodes demonstrated a dramatic reduction in metabolic activity with Sprycel [37].

There are many additional clinical trials that are testing efficacy of c-KIT inhibitors in melanoma (see Table S1). While final results from these studies are forthcoming, some interim results have been insightful. Results of an ongoing, Phase II trial of Gleevec therapy for metastatic melanoma patients were presented at ASCO's 2009 conference [38]. All patients in this trial had specific mutations in c-KIT and/or amplification of c-KIT as well as acral, mucosal, or chronic sun damaged melanoma (which often demonstrate c-KIT aberrations). Of twelve subjects, two had complete responses, two had partial responses, six had stable disease, and two progressed. Both of the complete responders had mutations as well as amplification. The differential responses were correlated with distinct c-KIT aberrations, suggesting that responsiveness to c-KIT inhibitors is genotype-specific (see Table 3).

**Table 3:** Treatment outcomes related to specific c-KIT aberrations.

No. of patients	Treatment outcome	c-KIT aberration
2	Complete response	L576P exon 11 mutation & amplification
2	Partial response	L576P exon 11 mutation & exon 13 mutations
6	Stable disease	Exon 13 mutations with or without amplification
2	No response	Mutations known to cause resistance to Gleevec in GIST

Interim results of another multi-institutional Phase II clinical trial were presented at the International Melanoma Congress in November 2009 [39]. This trial evaluated the efficacy of Gleevec on patients with mucosal, acral/lentiginous, or chronically sun-damaged skin. None of the 10 patients with wild-type/amplified KIT showed a clinical response, although two of these patients had stable disease for 6–7 months. Five of 10 patients with KIT mutations demonstrated a partial response to imatinib treatment, three of whom also had amplified KIT.

### Subtype 3

Subtype 3 harbors mutations in the G proteins, GNAQ and GNA11.

### Subtype 3.1 overview

Subtype 3.1 is characterized by a mutation in the GNAQ gene that affects codon 209 and which could drive constitutive activity of the MAPK pathway. GNAQ encodes the alpha subunit of a q class of heterotrimeric GTP binding proteins (Gq) that enable signaling from the cell surface to the protein kinase C (PKC) protein and finally the MAPK pathway. Mutations in GNAQ have been found in >85 of blue naevi, >50% of malignant blue naevi and ∼50% of ocular melanoma of the uvea [39]. While GNAQ is primarily viewed as relevant to uveal melanoma, anecdotal reports have found mutations in this gene in non-uveal melanoma patients as well. Studies so far have not identified other molecular aberrations that segregate with GNAQ.

### Subtype 3.2 overview

Subtype 3.2 is characterized by a mutation in the GNA11 gene that affects codon 209 and which, like GNAQ, could drive constitutive activity of the MAPK pathway. GNA11 was identified in a forward genetic screen in mice, along with GNAQ, looking for aberrant pigmentation symptoms in melanocytes. Like GNAQ, GNA11 encodes for the alpha subunit of a q class of heterotrimeric GTP binding proteins (Gq). Also like GNAQ, although GNA11 is primarily viewed as relevant to uveal melanoma, anecdotal reports have found mutations in this gene in non-uveal melanoma patients [40]–[41]. Mutations at codon 209 in GNAQ or GNAQ11 leading to constitutive activation can be detected through targeted sequencing.

### Potential therapeutic approach for subtypes 3.1 and 3.2

Several MEK inhibitors are currently in development and are potentially relevant to treatment of these subtypes.

### Subtype 4

Subtype 4 is associated with RAS gene abnormalities. Ras proteins are small GTPases that regulate cellular behavior in response to extracellular stimuli. Ras-regulated signal pathways control processes such as actin cytoskeletal integrity, proliferation, differentiation, cell adhesion, apoptosis, and cell migration via the MAPK and AKT/PI3K pathways.

Ras has many isoforms of which NRas and KRas are the most relevant to human cancer. These are mutated in an estimated 20–30% of all cancers [42]. While these isoforms are functionally similar, their roles may be tissue-specific [43]. For example, KRas aberrations are frequently found in pancreatic cancer [44] whereas HRas aberrations are frequently observed in bladder cancer [45]. The Q61R and Q61L NRAS mutations can be detected by targeted sequencing.

### Subtype 4.1 overview

Subtype 4.1 is characterized by mutations in NRAS, which are observed in approximately 20% of melanomas [46], [19].

### Potential therapeutic approach for subtype 4.1

Despite the wide breadth of knowledge implicating Ras in tumor initiation and promotion, Ras has not been successfully drugged. Two approaches have been considered. The first approach involves blocking farnesylation. However, a small clinical trial with a farnesyl transferase inhibitor failed to demonstrate efficacy in a melanoma cohort that was not selected based on NRas status [47]. A more stringently selected cohort may have been more responsive.

The second approach involves concurrently targeting downstream pathways such as the MAPK and AKT/PI3K pathways. This has proven efficacious in preclinical models [48], and will be tested clinically by Merck and Astrazeneca in the near future (NCT01021748: MK2206 and AZD6244 for solid tumors).

### Subtype 5

This subtype is characterized by abnormalities in the melanocyte development and survival pathway. Within this pathway the melanocyte transcription factor MITF (Microphthalmia-associated transcription factor) regulates development, differentiation, and maintenance of melanocytes [49]–[50]. MITF is activated by the MAPK pathway as well as the cAMP pathway and leads to transcription of genes involved in pigmentation (such as TYR and DCT) as well as cell cycle progression and survival. For example, MITF may contribute to increased Bcl-2 activity [51] and drive transcription of the cell cycle regulator, CDK2.

### Subtype 5.1 overview

This subtype is characterized by aberrations in MITF. A genome-wide analysis of copy number alterations in cancer identified MITF as an amplified locus in melanoma. Furthermore, MITF amplification correlated with decreased overall patient survival. In addition, MITF amplification was associated with increased resistance to chemotherapy [52] suggesting that it may serve as a good target for therapeutic intervention. Copy number aberrations in MIFT can be detected by commercial tests.

### Potential therapeutic approach for subtype 5.1

While MITF does not exhibit druggable activity, its expression was shown to be attenuated by multiple histone deacetylase (HDAC)-inhibitor drugs [53]. Building on this work, a clinical trial has recently begun accrual to evaluate efficacy of the HDAC inhibitor LBH589 on melanoma (NCT01065467- also see below). This study will also determine whether LBH589 effectively down regulates MITF in biopsy specimens of treated metastatic melanoma patients.

### Subtype 6

This subtype is associated with abnormalities in the AKT/PI3K signaling pathway which plays a pivotal role in modulating cellular functions such as proliferation, growth, survival, and metabolism in response to extracellular cues mediated by cell surface receptors and G-proteins. In the absence of external stimuli, PTEN generates the messenger phospholipid PIP2 by dephosphorylating PIP3. PIP2 cannot stimulate phosphorylation of the PI3K protein, which in turn maintains suppression of cell growth and division. The balance between PIP2 and PIP3 is maintained by PTEN (Phosphatase and TENsin homolog) and PI3K, a kinase that converts PIP2 into PIP3. Upon growth factor stimulation, PI3K is activated which leads to increase in PIP3 levels. PIP3 binds to Akt and then translocates to the plasma membrane where Akt is activated by phosphorylation. Activated Akt phosphorylates its substrates including the serine/threonine kinase mTOR which then phosphorylates S6 kinases (S6K) and inhibits 4E-BP, leading to increased protein translation as well as other targets that regulate cell division and apoptosis [54]. Relevant aberrations in PTEN and PI3K levels can be detected by IHC. Aberrations in AKT levels can be detected by copy number analysis.

### Subtype 6.1 overview

Subtype 6.1 harbors aberrations in PTEN, a lipid phosphatase that negatively regulates growth through the AKT/PI3K pathway. As described above, PTEN acts antagonistically with the lipid kinase, PI3K, to tip the balance between two signaling molecules, PIP2 and PIP3. Upon growth factor stimulation, PI3K is activated, increasing PIP3 levels which drives phosphorylation of AKT and downstream processes such as higher protein translation, cell division and reduced apoptosis.

Inactivation of PTEN is associated with a variety of cancers including glioblastoma, melanoma, and carcinomas of prostate, breast, and endometrial origins. Loss of (or reduced) PTEN protein is observed by immunohistochemistry (IHC) in 20–40% of melanoma tumor samples [55]–[57], [19]. Somatic PTEN point mutations and homozygous deletions are rare [58]. Consistent with its role in the AKT/PI3K pathway, 82% of specimens with PTEN loss had measurable increases in expression of pAKT [59].

PTEN dysregulation often occurs in conjunction with mutations in BRAF and this combination has been classified as subtype 1.2. The relative frequency of these subtypes is not clear though we hope to learn more based on patient reports and research studies. Subtype 6.1 specifically deals with dysregulation of PTEN in the absence of BRAF mutations which leads us to consider AKT/PI3K inhibitors as potential therapies for this subtype.

### Subtype 6.2 overview

Subtype 6.2 consists of aberrations in Akt, a protein kinase of the Protein Kinase B (PKB) family that plays a central role in coordinating cellular behavior with signals from a variety of extracellular pathways. As described above, Akt is regulated by the lipid signaling molecular PIP3, whose levels are determined by the interplay between PTEN and PI3K. Humans have three AKT genes: Akt1, Akt2, and Akt3 [60]. Akt1 is involved in apoptosis and protein synthesis, Akt2 is involved in glucose metabolism and Akt3 may be involved in several processes.

Akt has been implicated in many cancers including melanoma. More than 70% of primary and metastatic melanomas exhibit higher Akt activity as monitored by immunostaining against the Serine-473 residue of Akt [61], [56]. Additionally, the 1q43–44 genomic region that contains Akt3 is often amplified [62].

AKT dysregulation has been observed to occur in conjunction with mutations in BRAF, and this combination has been classified as subtype 1.3. The relative frequency of these subtypes is not clear, though we hope to learn more based on patient reports and research studies. Subtype 6.2 specifically deals with dysregulation of Akt in the absence of BRAF mutations, which leads us to consider AKT/PI3K inhibitors as potential therapies for this subtype.

### Subtype 6.3 overview

Subtype 6.3 is characterized by aberrations in PI3K, a lipid kinases that regulates growth through the AKT/PI3K pathway. As described above, PI3K acts antagonistically with the lipid phosphatase, PTEN, to tip the balance between two signaling molecules, PIP2 and PIP3. Upon growth factor stimulation, PI3K is activated which increases PIP3 levels and promotes phosphorylation of AKT and downstream processes such as increased protein translation, cell division and reduced apoptosis.

The PI3K protein family is divided into three classes and several subclasses based on primary structure, regulation, and *in vitro* lipid substrate specificity. Of these, Class Ia is the best understood, partly because of its role in cancer. These proteins are composed of a catalytic subunit (p110) and a regulatory subunit (p85).

PI3K expression is higher in malignant melanomas (as compared to blue nevi) and is correlated with a worse prognosis [63]. In contrast, activating mutations found in ∼1% of primary melanomas and comparative genomic hybridization did not reveal genomic amplification [59].

### Potential therapeutic approach for subtypes 6.1, 6.2 and 6.3

There are three potential targets for therapeutic intervention against this pathway: AKT, PI3K and mTOR. Both subtypes 6.1 and 6.3 could potentially be treated with all three classes of drugs, but subtype 6.2 is not expected to respond to PI3K inhibitors. There are several drugs in clinical development targeting all three, and a few drugs against mTOR that are currently approved for other cancer types (see Table S1). Results of these trials are anxiously awaited though they may be mixed because none of them are focused exclusively on patients with PTEN aberrations (or aberrations in the AKT/PI3K pathway). Even in a selected patient population results may be mixed. This was observed in a Phase I clinical trial investigating the impact of the mTOR inhibitor, Rapamycin, in PTEN-deficient glioblastoma; the drug proved effective in suppressing disease progression in some patients but appeared to accelerated disease in others [64].

Pending trial results, a few case reports have emerged suggesting efficacy of Rapamycin in conjunction with the chemotherapeutic drugs carboplatin and paclitaxel in melanoma [65]. This theme has also been observed across several cancers including ovarian, breast, and pancreatic carcinomas and points to a universal role of this pathway in driving chemoresistance [66]. Several clinical trials listed below are investigating specific combinations of mTOR inhibitors and chemotherapy drugs in the treatment of melanoma.

### Subtype 7

This subtype is characterized by aberrations in the G1/S Cyclin/CDK pathways. CDKs belong to a family of protein kinases that control cellular proliferation by phosophorylating proteins involved in the regulation and mechanics of processes such as growth, DNA replication, and mitosis. The cyclin proteins are regulatory subunits that bind and activate the CDKs that bear catalytic kinase activity.

Several distinct types of cyclins and CDKs have been identified and appear to drive distinct stages of the cell cycle. For example, Cyclin D/CDK4 complexes drive passage from the pre-replicative (G1) to the DNA duplication (S) phase, the Cyclin E/CDK2 complexes drive DNA duplication, and the Cyclin B/CDK1 complexes drive entry into mitosis. In addition to the cyclins, the cell cycle is influenced by numerous inhibitors (such as p16INK4) and activators that ensure mutual dependence of DNA replication and mitosis as well as coupling to extracellular signals [67]. Targeted sequencing and Comparative Genomic Hybridization (CGH) assays are available for ARF/INK4A, copy number analysis for CDK4, and IHC for CCND1/Cyclin D.

### Subtype 7.1 overview

This subtype is associated with aberrations in ARF/INK4A, which encodes for p16INK4, a cell cycle regulator, and p14ARF, a regulator of the p53 pathway (http://www.ncbi.nlm.nih.gov/gene/1029). p16INK4 binds to and inhibits CyclinD & CDK4/6 complexes which suppresses progression from G1 to S transition. p14ARF binds to, and inhibits, MDM2 which leads to stabilization of p53.

The ARF/INK4A gene is a key susceptibility locus for familial melanoma [68], and is also often somatically mutated in melanoma. ARF/INK4A mutations in melanoma typically occur in the p16INK4 gene either alone or in combination with p14ARF, suggesting that p16INK4 is the relevant tumor suppressor. p16INK4 is deleted in approximately 50% of melanomas and inactivated by point mutations in about 10% [69]. In addition, this gene is often transcriptionally silenced by promoter hypermethylation [69]–[70]. Reduced p16INK4 levels correlate with disease progression [71]–[72] and poor prognosis [70]. However, some families harbor mutations only in p14ARF suggesting a role in melanoma progression [74]–[75]. Mouse studies indicate that either is sufficient for melanoma predisposition and that they might, in fact, cooperate to drive disease progression [76]–[77].

### Subtype 7.2 overview

This subtype is characterized by aberrations in CDK4, which drives passage from G1 to S phase in complex with Cyclin D by phosphorylating and inactivating the retinoblastoma protein (RB) inhibitor. CDK4 amplification is relatively common in acral and mucosal melanomas [26], [78]. Additionally, a substitution of Cysteine for Arginine at the 24th codon of CDK4 is observed in a small percentage of melanoma-prone families [79]. CCND1/ Cyclin D amplification is observed in approximately 4% of melanomas [26], [78], [80].

### Subtype 7.3 overview

Subtype 7.3 is characterized by aberrations in Cyclin D, which drives passage from G1 to S in complex with CDK4 and CDK6. Cyclin D is commonly found to be aberrant in cancer in terms of mutation, amplification, and/or overexpression. Overexpression has been observed in mantle cell lymphoma, non-small cell lung cancer and carcinomas of breast, head and neck, and esophagus. Amplification of the Cyclin D gene has been observed in tumors such as head and neck carcinomas, pituitary tumors, esophageal squamous cell carcinoma, and breast cancer [81].

In melanoma, genomic amplifications of Cyclin D are primarily found in acral lentiginous melanoma (∼44%), and to a lesser degree in other types (11% for lentigo maligna and 6% for superficial spreading melanoma) [82]. Antisense-mediated knockdown of CCND1 triggers apoptosis *in vitro* and shrinkage of xenografts in mice [82], suggesting that Cyclin D plays a role in melanoma tumorigenesis and so may be a good target for therapeutic intervention.

### Potential therapeutic approach for subtype 7

Any therapeutic strategy for subtype 7.1 will likely depend on the specific mutations within individual tumors. Tumors with mutations that only affect p16INK4 could potentially be addressed with inhibitors of CDK4/6. There are currently no validated therapeutic options for tumors with only p14ARF mutations. For subtypes 7.2 and 7.3, several CDK4 inhibitors are currently in development for a variety of cancer types. UCN-01 is currently involved in ongoing Phase II trials for metastatic melanoma.

### Subtype 8

This subtype is associated with aberrations in the p53-regulated intrinsic cell death pathway. This pathway is initiated by p53 in response to cellular stress such as growth factor deprivation, hypoxia, cell detachment, or DNA damage [83] and results in the activation of the Bcl-2 family of proapoptotic genes. The Bcl-2 pathway contains activators and inhibitors of apoptosis that together influence the fate of a cell. IHC can detect aberrations in the level of BCL-2, and targeted sequencing can detect P53 mutations.

### Subtype 8.1 overview

Subtype 8.1 is characterized by aberrations in Bcl-2, a key inhibitor of cell apoptosis. The Bcl-2 family of proteins contains both pro- and anti-apoptotic members that regulate apoptosis via a delicate balance [84]. The Bcl-2 pathway is activated via p53 in response to cellular stress such as growth factor deprivation, hypoxia, cell detachment, or DNA damage. Several lines of evidence point to a key role of this pathway in melanoma pathogenesis: 1. BCL-2 is overexpressed in melanoma [85]–[86]: Factors including NRas [87] and MITF may also contribute to increased Bcl-2 activity [51]. 2. Anti-sense suppression of Bcl-2 leads to decreased melanoma cell survival and increased sensitivity to chemotherapy [88]–[89], [51]. 3. Bcl-2 overexpression reduced apoptosis and sensitivity of melanoma cells to pro-apoptotic stimuli.

### Subtype 8.2 overview

Subtype 8.2 is characterized by mutations in the tumor suppressor, p53. p53 is mutated in greater than half of all human cancers but in only about 10% of melanomas [90]. However, studies in mice and *in vitro* studies indicate that the p53 pathway contributes to melanoma. In particular, it appears that the p53 pathway can be suppressed by directly inactivating p53 or by modulating the p53 inhibitor, p19ARF. Consistent with this, there are sporadic examples of human melanoma cases with mutated p19ARF. p53 also appears to play a role in resistance to chemotherapy because apoptosis is considered to be the primary mechanism by which chemotherapeutic drugs drive tumor cell death [91]. Mutant p53 cell lines appear to be refractory to drugs like cisplatin, vincristine and camptothecin [92]–[93].

### Potential therapeutic approach for subtype 8

There are currently no drugs approved or in trials that could be therapeutic for this subtype of melanoma. However several, including YM155, ABT-737, AT-101, and TW37, are in preclinical development. One anti-apoptotic agent, Oblimersen, an anti-sense agent targeted at nuclear Bcl-2 has exhibited mixed results in melanoma. Survival results from the AGENDA Phase III trial of Oblimersen are awaited. However, lack of tumor Bcl-2 expression fails to confirm an *in vivo* mechanism of action [94]. Recently, results of a Phase I/II study evaluating the effect of a combination of Oblimersen, Temozolomide, and Abraxane on patients with advanced melanoma (NCT00518895) showed increased overall survival as compared to the previous trial. In addition, phenotypic changes were observed in Bcl-2 and related family members and correlated with treatment outcomes [95].

## Discussion

This paper describes a 'Molecular Disease Model' (MDM) for melanoma. Given the evolving state of knowledge, we anticipate that the current model will be revised with new clinical and scientific findings. To support the efficient use and dynamic updating of this MDM, we have posted it in hyperlinked “semantic wiki” format here: http://mmdm.cancercommons.org/smw/index.php/A_Melanoma_Molecular_Disease_Model.

We intend to regularly update this online version of the model as new results appear in the literature.

Our goal in putting the melanoma MDM online in this way is to facilitate the rapid distribution and updating of this important and timely information. In addition, applications can be developed by directly utilizing the model's semantic content.

## Supporting Information

Table S1 **Drugs, their manufacturer and putative targets for each subtype, as well as the relevant clinical trials and their status.**
(XLS)
